# A Case of Polymyositis Associated with Cytomegalovirus Infection in a Patient with Hashimoto’s Thyroiditis

**DOI:** 10.3390/life13122331

**Published:** 2023-12-12

**Authors:** Ergeta Ktona, Blerta Budani, Ioannis Kostas-Agnantis, Alma Idrizi

**Affiliations:** 1 Rheumatology Department, University Hospital Center, 1001 Tirana, Albania; 2 Faculty of Technical Medical Sciences, University of Elbasan, 3001 Elbasan, Albania; blerta.budani@mail.huji.ac.il; 3 Orthopaedic Department, University Hospital of Ioannina, 451 10 Ioannina, Greece; ioanniskostas@hotmail.com; 4 Nephrology Department, University Hospital Center, 1001 Tirana, Albania; alma_idrizi@yahoo.com

**Keywords:** polymyositis, cytomegalovirus infection, hashimoto’s thyroiditis, association, coexistence, autoimmune diseases

## Abstract

Polymyositis is a rare condition with an unknown etiology occurring more frequently in adult women. There is a lack of evidence on the coexistence of PM and CMV infection in a patient with hypothyroidism due to Hashimoto’s Thyroiditis. However, the growing occurrence of both CMV infection and the simultaneous occurrence of autoimmune diseases points out a relationship, while the association direction remains unclear. Case outline: A 32-year-old woman recently treated for HT hypothyroidism was admitted to the hospital two weeks after being treated for common flu by the family doctor, complaining about a worsening condition with muscle pain, weakness, frequent falls, and fatigue. The first tests showed a normalized thyroid function, with elevated values of troponin and serum creatinine kinase (CK). The immunological tests revealed the presence of a high titer of CMV IgG antibodies and raised levels of CMV DNA. Pelvis MRI images demonstrated markedly elevated signals on the STIR sequences in the pelvis, thighs, and calves, indicating active and severe multifocal myositis. The diagnosis of PM was confirmed with the muscle biopsy on day 7 of hospitalization. The patient showed significant improvements within two weeks after the medical therapy and physiotherapy.

## 1. Introduction

Idiopathic inflammatory myopathies (IIMs) also known as myositis are rare inflammatory disorders that affect the skeletal muscles and include dermatomyositis (DM), polymyositis (PM), overlap myositis (OM), which includes anti-synthetase syndrome (ASS), and inclusion body myositis (IBM), and necrotizing myopathy (NM) caused by an immune response [1]. PM as a subset of IIMs, is manifested by progressive muscle weakness, electromyography (EMG) alterations indicative of myopathy, biopsy evidence of inflammatory infiltrates, and changes in muscle enzyme levels [2]. It is a remarkably uncommon entity, affecting more women than men and rarely found in children [3]. However, the diagnosis and treatment of all myopathies including PM is challenging, as it requires the involvement of an interdisciplinary team of rheumatologists and/or neurologists, pulmonologists, cardiologists, dermatologists, physiotherapists, etc. The consensus among international experts is the EULAR/ACR new classification criteria for diagnosis of all types of myositis which is built upon the traditional Boran Bohan and Peter criteria and includes (i) a thorough clinical assessment including the pattern of weakness and paraclinical parameters such as MRI and CK, etc.; (ii) a broad assessment of autoantibodies; and (iii) a muscle biopsy with detailed histological and immunohistological analysis that allows subtyping of the different pathological entities [4].

The diagnosis becomes more complicated in the presence of another autoimmune condition. Autoimmunity is a normal aftermath, which when aberrated can result in loss of self-tolerance leading to autoimmune diseases. It covers a wide range of diseases from systemic diseases including Systematic Lupus Erythematous (SLE) and PM to organ-specific diseases like Hashimoto’s Thyroiditis (HT). These diseases are characterized by the generation of a large range of autoantibodies targeting multiple autoantigens. Despite the fact that the etiology of these diseases is not completely understood, environmental, genetic, and immunological factors contribute to the phenomenon of their etiology, referred to as the “mosaic of autoimmunity” [5].

Although it is generally acknowledged that there is an association between autoimmune thyroid disorders and systemic autoimmune diseases [3,4,5,6,7], there are very few reports available confirming a possible relationship between PM and HT [8,9,10]. While the etiology of autoimmune diseases is not completely clear, PM is thought to be triggered by viruses such as coxsackie viruses, enteroviruses, retroviruses, parvoviruses, human T-lymphotropic virus (HTLV), and human immunodeficiency virus (HIV) [11]. Cytomegalovirus (CMV), part of the herpesvirus family is acquired in the first years of life and is carried by 40–70% of the world’s population [12,13]. Some evidence has shown that herpesviruses such as Epstein Barr virus are linked to several autoimmune diseases including PM and HT [14]. However, there is a lack of evidence to support the hypothesis on the association between PM and CMV infection. We describe a 32-year-old woman newly diagnosed with HT hypothyroidism, presented with PM occurring simultaneously with CMV infection.

## 2. Case Presentation

A 32-year-old woman diagnosed and treated for HT with hypothyroidism for less than one year was presented to the family doctor complaining about progressive muscle pain, fatigue for about a week, and a two-week history of daily sub-febrile temperature of 37.5° Celsius. The patient worked as an engineer and reported to be a nonsmoker, with no previous personal or family history of thyroid disorders, or autoimmune diseases, and without known allergies. The family physician diagnosed her with common flu, and she was given only paracetamol and vitaminic therapy for ten days. During the following days, the situation aggravated with progressive bilateral lower extremity weakness, walking difficulties, recurrent falls, fatigue, and disability to perform daily activities. After 2 weeks, she was admitted to the rheumatology department for further examinations. During the physical examination, the initial observation was symmetrical muscle weakness, beginning in the proximal lower extremities and later extending to the upper extremities (++++) with no involvement of the esophageal muscles. Additionally, the neck flexors were weaker than the extensors. The patient reported difficulties in combing her hair, problems with elevating her arms over the head, and frequently having a feeling of dyspnea. There were no apparent skin lesions or other clinical signs of Lupus Erythematosus. Lung auscultation revealed low-sounding crackles over basal parts.

The first tests showed a normalized thyroid function with free thyroxine (free T4) level of 19.2 pg/mL (normal range 12.0–22.0 pg/mL), thyroid-stimulating hormone (TSH) 1.87 mlU/L (normal range 0.3–4.5 mlU/L) with elevated troponin 4.95 ng/mL (normal range 0.0–3.37 ng/mL) and serum creatinine kinase (CK) levels 1038 U/L (normal range < 195 U/L, ERS 5 mm/h. Blood test, red blood cell (RBC) 3.9 × 10^6^ (normal range 4–5 × 10^6^), white blood cell (WBC) 3.4 × 10^3^ (normal range 4.00–11.00 × 10^3^), hemoglobin (HGB) 10.8 g/dL (normal range 12.0–14.0), HCT 33.2% (normal range 35.0–50.0%), platelets (PLT) 116 × 10^3^ (normal range 150–400 × 10^3^), and erythrocyte sedimentation 45 mm/h. AST 450 U/L (normal range < 38 U/L), ALT 280 U/L (normal range < 32 U/L). LDH 390 U/L (normal range < 135–225 U/L) Aldolase 65 (normal range < 8 U/L). PCR 12 (normal range 0–5 U/L), and Myoglobin 185 mcg/L (normal range 5–70 mcg/L).

The immunological tests revealed the following results: IgA 1.38, IgG 7.1, IgM 76, C3 complement 12.7 (90–180 mg/dL), C4 complement 22 (10–40 mg/dL), and normal Rheumatoid Factor (RF) < 10 IU/mL (<15 IU/mL), Anti CCP < 7 (normal range < 17 U/mL). In addition, Extractable Nuclear Antigen (ENA) screening resulted in positive, Ro (SS-A) antibody positive, Anti-SRP-54 weak positive, Sm antibody positive, Proteinase 3 (PR3) antibody < 0.2 (normal range 0–1 AI), and Myeloperoxidase (MPO) antibodies < 0.2 (normal range 0–1 AI). While La antibody, RNP antibody, Jo-1 antibody, Scl-70 antibody, Ribosomal antibodies, myocardial antibody, and cardiolipin antibody were all negative. Results from other laboratory tests were as follows: TB gamma interferon negative, HBsAg negative, anti-HBc negative, HCV IgG negative, HIV 1, and HIV 2 antibody negative, antiphospholipid antibodies negative, and Mycoplasma pneumonia antibody negative. Laboratory tests for Toxoplasma Gondi and EBV showed signs of infection at some time but no evidence of recent infection. CMV IgM was negative, and only CMV IgG was detected. CMV DNA was found to be 209,512 IU/mL (normal range < 200 copies/mL) and Anti DNA was negative. There was also no presence of mucocutaneus signs or joint and renal involvement. Due to the high levels of CMV DNA, in consultation with the virology team, it was decided to start the treatment with valganciclovir 900 mg twice daily for 21 days, and then 900 mg per day.

To look for changes in the organs within the pelvis, we performed a pelvic ultrasound transabdominal study which showed a retroverted uterus, appearing in normal shape and size (8 cm long, 5 cm across and 4.8 thick cm) with no obvious adnexal masses, cysts, or free fluid. Images from pelvis MRI demonstrated an extensively increased signal on the STIR sequences within the visualized pelvis, thighs, and calves within multiple muscle groups consistent with active and severe multifocal myositis.

A cardiac echo demonstrated a normal-sized left ventricular cavity, normal LV systolic function by ejection fraction, no valvular dysfunction, and no pericardial effusion. Additionally, a PET scan was organized but it was refused by the patient. EMG finding was abnormal and revealed the presence of fibrillation potential and positive sharp waves suggestive of myopathy. We then performed a cardiac MRI which showed the following: nondilated LV with normal systolic function, no LVH, normal RV Size, and systolic function, patchy non-ischemic fibrosis in the basal to mid inferior segment (borderline elevated native T1 values, normal T2 readings), and small bi basal pleural effusion, right > left. There was no evidence that the myocardium had been involved, which is consistent with the clinical picture of inflammatory myositis. Troponin T was elevated but presented more skeletal muscle release than cardiac involvement.

A biopsy of the right biceps muscle was performed on day 7 of admission to the hospital and demonstrated mild and non-specific changes with noticeable myocyte phagocytosis. The structural proteins investigated were normal. The diagnosis was confirmed by the detection of the presence of anti-SRP autoantibodies. All the examinations were performed during the first days of her recovery, a second biopsy was suggested but it was refused by the patient.

According to the clinical traits, the laboratory findings, and based on EULAR/ACR criteria [5], the woman was diagnosed with PM and CMV infection concomitantly.

We, therefore, started the treatment with an IV methylprednisolone course, immunoglobulins 20 g for 5 days, MTX 15 mg s/c weekly, folic acid 5 mg once weekly, multivitamin 1 tab daily, 60 mg prednisone weaned down now to 30 mg, Bisoprolol 5 mg daily, Valganciclovir 900 mg twice daily, Lansoprazole 30 mg daily, Levothyroxine 100 mg, Fortisip 1 mg twice daily, Ivabradine 5 mg twice daily, and Rituximab first doze followed by the second dose two weeks later.

Supplementing to medical therapy, The patient also attended three times a week physical therapy sessions. These exercises have focused on her proximal and core control with bed, chair, and standing exercises at a handrail. After the start of the treatment along with physiotherapy, there were improvements in her clinical features, in the number of steps she could complete and her trunk control despite being hampered by her fatigue. The laboratory tests showed that troponin and CK levels gradually dropped. After the second dose of Rituximab, the patient showed independent mobilization at a slow pace and was able to sit and stand from a chair and bed. The future treatment plan is as follows: Rituximab in the next 6 months, Methotrexate needs to be continued for 2–3 years minimum with regular monitoring (blood test once every 1–2 months for FBC and LFTs), and Prednisolone will continue at 30 mg for 2 weeks and it will be reduced with 5 mg every 2 weeks to result at 10 mg dose after 12 weeks. Prednisolone 10 mg was then recommended to be followed for 6 months.

## 3. Discussion

Our patient was a 32-year-old woman diagnosed and treated for HT hypothyroidism, who developed PM and CMV infection simultaneously. To the best of our knowledge, this is the only case report of PM associated with CMV infection in a patient with HT. There were some difficulties in interpreting the examination results and various challenges in managing the complexity of the diseases’ coexistence in this case.

The observed features initially suggested the possibility of a limb girdle dystrophy; however, the immune-mediated necrotizing myopathy (IMNM) was excluded as this is usually responsive to treatment. IMNM is characterized by acute muscle weakness, CK levels higher than 3000 IU/L, but with no concomitant inflammation, and frequently no enhanced production of MHC Class I proteins as this case presented. While clinical findings such as low C3, anti-SS A, and anti-SM positivity, and pleural effusion were suggestive of SLE diagnosis, this was excluded because the patient did not fulfill the EULAR/ACR diagnostic criteria. Additionally, the diagnosis of viral myositis was also excluded as IgM was negative and IgG positive, indicating a previous infection, and the presence of autoantibodies was revealed. Furthermore, based on a recent article on the role of SARS-CoV2 and other viral infections on neuromuscular disorders [15], considerations were given to the diagnosis of Guillain Barré syndrome (GBS), as a classic complication of a viral infection. However, this diagnosis was also ruled out as the patient did not present symptoms of acute flaccid paralysis, respiratory symptoms and laboratory findings to confirm, the diagnosis of GBS. The lumbar puncture data did not support the diagnosis of GBS.

This led us to consider the hypothesis of the coexistence of PM and CMV infection in this HT patient. Limited evidence suggests a possible association between PM and HT, between PM and CMV infection, and between CMV infection and HT. Although the literature confirms CMV as a notorious agent for autoimmune diseases, we found only one case report on polymyositis associated with primary CMV infection [16]. The study suggested two potential interpretations of the physiopathology of PM: PM triggered by the indirect role of CMV infection, and viral myositis, which resulted from muscle cell CMV infection. With regard to the second hypothesis, the inflammatory cell infiltrate in muscles found in our patient which are typically found in patients with PM, rule out the presence of viral myositis. Therefore, the first possible explanation which considers CMV infection as a trigger for PM is more likely. Moreover, the lack of the presence of CMV IgM and the IgG seropositivity demonstrates a primary CMV infection in our patient and supports the role of a trigger. As for the possible mechanisms, two explanations were proposed: immunological cross-reactivity as a result of molecular mimicry and virus-induced expression of histocompatibility complexes [13].

From the literature search, we found two case reports on the link between PM and HT. Sung and coworkers [10] described the case of a 20-year-old woman who was diagnosed with polymyositis and Hashimoto’s thyroiditis concomitantly and a study by Wang and coworkers [9] presented the case of a 45-year-old woman with hypothyroidism who developed polymyositis. Even though the coexistence of PM and autoimmune thyroid diseases (AITD) is not well documented there seems to be a potential common pathogenic mechanism. The potential mechanisms that have been postulated include the following: (1) common environmental factors such as a drug, a chemical, or a virus triggering both AITD and polymyositis in the genetically susceptible host [9,17]; (2) a genetic link between antithyroid autoimmunity and the susceptibility to autoimmune disease [9,10]; (3) molecular mimicry between PM and disease-specific epitopes [10]; (4) cross-reactivity of antithyroid antibodies or autoreactive T cells with other organs or other autoantibodies [9,10,16]; (5) immunomodulatory effects of antithyroid antibodies [18,19]; and (6) cytokine imbalance [10]. Both studies provide evidence that these two diseases may overlap and show that PM diagnosis could precede or parallel hypothyroidism.

Regarding the relationship between CMV and HT, some evidence implies that CMV is a causal agent for thyroiditis [13,20] and others have shown the presence of active CMV replication in patients with HT [12]. Larouche and coworkers [21] reported the case of a 49-year-old immunocompetent woman who experienced an episode of cytomegalovirus-induced mononucleosis before developing thyroiditis. In a review, Freeman, R.B. [12] states that CMV replication has been found in many areas of inflammation in several autoimmune diseases including HT However, the review does not address the question of whether inflammation is caused by CMV replication or vice versa.

Moreover, there is growing evidence that suggests that CMV infection is associated with autoimmune diseases. CMV is part of the herpes virus family, and it is usually acquired in the first few years of life via direct contact with other individuals’ infected body fluids. After the initial immune response, the virus becomes dormant and may reactivate in cases of diminished immunity. While in most cases CMV reactivation may be asymptomatic, in many other cases, particularly in immune-compromised people, may have severe consequences. However, the literature shows that CMV infection plays a critical role in autoimmunity even in immunocompetent individuals. A recent review by Gugliesi and coworkers [21] suggests the existence of an interaction between CMV and the immune system, supported by the fact that the viral infection has been found in several autoimmune diseases, such as neurological, enteric disorders, and metabolic diseases, and rheumatological diseases in particular. The study also postulates that the documented mechanisms of this interplay include inflammation, molecular mimicry, and non-specific B-cell activation. Furthermore, based on findings from the literature, the authors suggest that autoimmune diseases and CMV infection reciprocally affect each other. On the one hand, primary or secondary CMV infection can cause persistent type I inflammation throughout the body, which encourages autoimmunity and thus causes autoimmune disorders. On the other hand, autoimmune flares can also trigger CMV reactivation [21,22].

In our case, based on the currently available literature summarized in this report it is reasonable to support the theory of CMV infection as a shared environmental causal factor for PM and HT. Additionally, we suggest that the proposed etiopathogenic mechanism of molecular mimicry was found as a potential explanation for the relationship between PM and HT and between PM and CMV infection, and the link between CMV and HT is more likely to have caused the overlap over these three diseases. Molecular mimicry is a concept in immunology and autoimmune diseases research that describes the mechanism that is used to explain how the immune system may cause the development of antibodies that attack the self since viruses, bacteria, and their hosts all contain structurally similar or identical antigens. The four different forms of molecular mimicry include the following: (1) complete identity at the protein level between a microorganism and its host; however, the protein is not encoded by the microorganism; (2) homology at the protein level between a microbe and its host, of a protein which is encoded by the microbe; (3) similarity at the level of epitopes or amino acid sequences between the environmental agent or the microorganism and its host; and (4) structural similarities between the microbe or environmental agent and its host. For instance, the human cytomegalovirus (CMV) envelope contains CD13 (aminopeptidase N) molecules, thus presenting the first form of molecular mimicry [22]. This cross-reactivity between self and viral antigens where structural parts of the pathogen such as viral epitopes “imitate” or “stimulate” the molecules of the host is thought to be responsible for the development or exacerbation of several autoimmune diseases. Yet, there are four criteria that need to be fulfilled in these cases including the following: (1) similarity between a host epitope and an environmental or microbial agent’s epitope; (2) detection of antibodies, B or T cells that are cross-reactive with both epitopes in autoimmune disease patients; (3) the epidemiological connection between the onset of an autoimmune illness and exposure to an environmental chemical or microorganism; and (4) reproducibility of autoimmunity in an animal model after exposure to the environmental toxin, infection with the microorganism, or sensitization with the epitopes [23]. However, testing the fulfillment of the criteria of molecular mimicry was not part of the study’s objectives, as this study aimed at presenting a unique case of viral and autoimmune diseases overlapping and shedding light on the potential interpretations of the common etiopathology. Nevertheless, we do not exclude any of the other possible interpretations, leaving the question open to whether CMV infection can play a role as a trigger for autoimmune diseases or not.

In conclusion, our findings suggest a potential interplay between the immune system and CMV. Although there have been significant efforts to study the connection between CMV infection and autoimmunity, it is still not well documented if CMV infection induces autoimmune disorders or is a by-product that is associated with the perpetuation of autoimmune diseases. Further research is needed to elucidate this relationship.

## Data Availability

Available from the corresponding author on reasonable request.
